# A Clinical, Radiological and Histopathological Review of 74 Ossifying Fibromas

**DOI:** 10.1007/s12105-022-01522-w

**Published:** 2023-01-09

**Authors:** L. H. C. Collins, N. F. T. Zegalie, I. Sassoon, P. M. Speight

**Affiliations:** 1 School of Clinical Dentistry, University of Sheffield, 19 Claremont Crescent, Sheffield, S10 2TA UK; 2 Present Address: Head and Neck Pathology, Guy’s Hospital, Floor 4 Tower Wing, London, SE1 9RT UK; 3 Department of Computer Science, Brunel University, Kingston Lane, London, UB8 3PH UK

**Keywords:** Ossifying fibroma, Cemento-ossifying fibroma, Histology, Radiology, Oral pathology, Odontogenic tumours

## Abstract

**Background:**

Ossifying fibroma (OF) is a fibro-osseous lesion of the jaws and craniofacial bones. Accurate diagnosis can be challenging due to significant overlap of clinicopathological features. This study aimed to evaluate the clinical, radiological and histological features that can aid in diagnosis and identify characteristics that allow categorisation into the three subtypes: juvenile trabecular, psammomatoid and cemento-ossifying OF.

**Methods:**

A total of 74 cases of OF were systematically reviewed for their principle features. Of these, 46 cases were evaluated for their radiographic features including size, location and relationship to the teeth. Histological assessment and stereological point counting were performed in 69 cases to assess the pattern, type and proportion of calcification, the nature of the stroma, the border of the lesion and the presence of secondary changes. Fisher’s exact test and Chi-squared tests were used to determine associations between clinicopathological parameters and maxillary, mandibular, odontogenic, non-odontogenic and psammomatoid or trabecular lesions.

**Results:**

OF showed a female predilection (F: M; 2:1) and a slight bimodal age distribution with peaks in the second (23%) and fourth decades (27%) (Mean age: 32.4 years). 83% of cases presented as an intra-oral swelling, with the mandible being the most common site (73%). Histologically, a range of morphological patterns were seen, with 50% of cases showing mixed trabecular and psammomatoid features. However, there were no significant differences between the variants of OF in terms of age, gender or histological features.

**Conclusion:**

Histological features of OF cannot be used to differentiate between the subtypes.

## Introduction

### Background

Ossifying fibroma (OF) is the most common fibro-osseous lesion of the oral and maxillofacial regions. This benign neoplasm exhibits progressive enlargement and bony expansion that can result in asymmetry, facial disfigurement and malocclusion [1–6]. There have been many changes to terminology over the years, including terms such as periodontoma, cementifying fibroma and ossifying-odontogenic fibroma, that give reference to their frequent association with teeth and the presence of cementum-like material. The 1992 WHO classification of odontogenic tumours preferred the term ‘cemento-ossifying fibroma’ and was the first to recognise a more rapidly growing subset of these lesions referred to as ‘juvenile aggressive ossifying fibroma’ [7]. Later, in the 2005 WHO classification, this subset was further refined to juvenile trabecular ossifying fibroma (JTOF) and juvenile psammomatoid ossifying fibroma (JPOF) largely based on their pattern of calcification, age of onset, location and recurrence rate [8]. All other lesions were referred to as ‘ossifying fibroma’ on the basis that cementum and bone have the same composition without distinction between the two when not associated with the root of a tooth.

In 2017, the terminology changed once more, with the WHO consensus panel agreeing to restore the term ‘cemento-ossifying fibroma’ (COF) because it better reflected the fact that these lesions arise within the tooth-bearing areas of the jaws and are benign mesenchymal odontogenic tumours that probably arise from the periodontal ligament [9]. Thus three types of ossifying fibroma were recognised; the cemento-ossifying fibroma as a type of odontogenic tumour, and the JTOF and JPOF which were considered non-odontogenic and classified under benign fibro- and chondro-osseous lesions.

Some believe that because treatment by surgical excision is required for all types of lesion, separation into odontogenic and non-odontogenic variants is not warranted [10], while others feel that odontogenic and non-odontogenic lesions should be clearly separated into distinctive entities to reflect their aetiology. Additionally, the misleading term ‘juvenile’ should be dropped since there is no clear distinction between juvenile and adult forms [11]. In the latest (2022) WHO classification, the term cemento-ossifying is still used for a variant that arises in the tooth-bearing areas of the jaws and is thought to be odontogenic origin [1]. The new classification also removes the term “juvenile” from the psammomatoid variant because of its wide age distribution [3]. Thus, the WHO recognises three variants: cemento-ossifying fibroma, juvenile trabecular ossifying fibroma and psammomatoid ossifying fibroma [1–3].

### Cemento-Ossifying Fibroma

Cemento-ossifying fibroma has a peak incidence in the third and fourth decades of life and exclusively found in the tooth-bearing areas of the jaws, commonly affecting the pre-molar and molar region of the mandible [12, 13]. Grossly, they are well circumscribed, mimicking their radiographic appearance, and often shell out of the surrounding normal bone. Their histological appearance is a variable admixture of a fibrous connective tissue stroma containing foci of mineralisation, which can be variable in morphology and frequently comprises cementum-like tissue.

### Juvenile Trabecular Ossifying Fibroma

As the name suggests, the juvenile variant is seen predominantly in children and is rarely seen over the age of 15 [2, 14]. JTOF features slender elongated immature trabeculae of bone, with plump osteocytes, often resembling osteosarcoma [15–19]. Like COF, this variant may also occur in the gnathic bones, but are located in the non-tooth-bearing areas, most often in the posterior/ramus region of the mandible or associated with the maxillary antrum.

### Psammomatoid Ossifying Fibroma

POF is reported in a wide age range from 3 months to 72 years and although they may be found in the jaws, they have a predilection for the craniofacial skeleton and are found in the bones of the paranasal sinuses [3, 15–19]. POF is characterised by multiple mineralised psammomatoid bodies or ‘ossicles’ set within a cellular stroma.

Despite a now well-established classification, ossifying fibromas still represent a considerable diagnostic challenge for even the most experienced clinician and pathologist, due to the apparent clinical, radiological and histological overlap between the variants. This diagnostic challenge is further complicated by the paucity of studies comparing odontogenic and non-odontogenic subtypes.

## Aim

The aim of this study was to assess the histopathological features of ossifying fibromas of the maxillofacial region and to correlate these to the clinical and radiological features to identify characteristic differences that may allow classification into the three subtypes: COF, JTOF and POF. We hypothesise that, in addition to their location in the non-tooth-bearing areas of the jaws, non-odontogenic OF lesions show specific age, gender, size and histological features when compared to the odontogenic OF variants.

## Materials and Methods

### Sampling

**Fig. 1:**
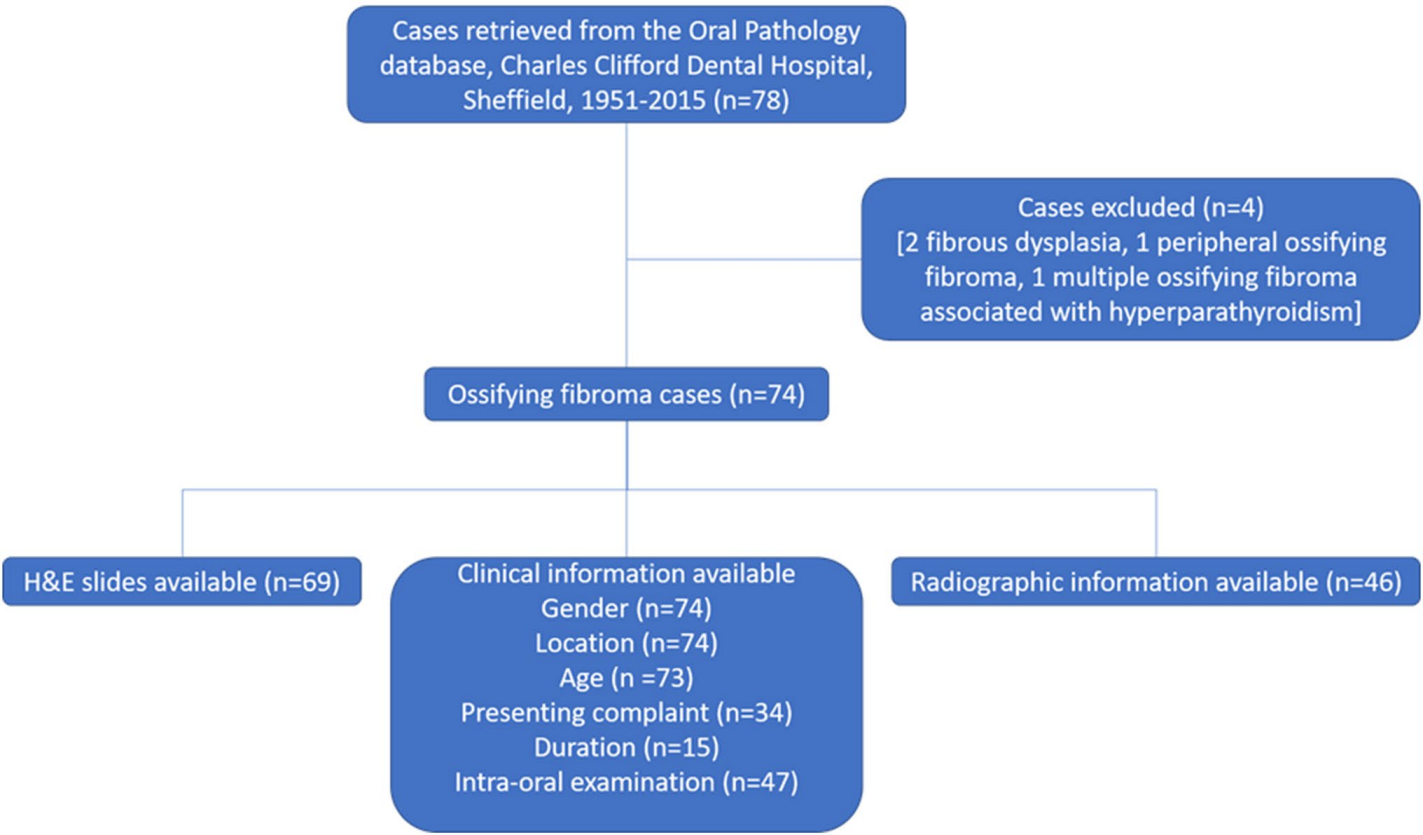
Case selection of ossifying fibromas from the archives of the Pathology Department, Charles Clifford Dental Hospital, Sheffield, covering the period between 1951 and 2015

A total of 78 patients were identified from the archives of the Oral Pathology Department, Charles Clifford Dental Hospital, Sheffield, covering the period between 1951 and 2015. Clinical and histological features of each case were reviewed and four patients were excluded from the study because they had a diagnosis of fibro-osseous lesions other than OF (*n* = 2), peripheral OF (*n* = 1) or a fibro-osseous lesions associated with hyperparathyroidism (*n* = 1). A total of 74 patients (Fig. 1) were finally included in the study. Four patients also had recurrent lesions, but only the first presenting lesion was used, giving a total of 74 OFs.

### Clinical and Radiological Details

Clinical and radiological parameters were recorded as shown in Fig. 1 and Table 1. Gender, location and age were known for all but one patient. Radiographs were available for review for 46 cases. Lesions were classified as odontogenic or non-odontogenic according to the relationship of the centre of the lesion to the ID canal or to the maxillary sinus floor. In the mandible, lesions were defined as odontogenic if they were in the tooth-bearing areas and the lesion was located above the ID canal (i.e. within the alveolar bone). Often the ID canal was seen to be deflected downwards below the lesion. In the maxilla, lesions were considered odontogenic if the lesion was located entirely within the maxillary alveolus below the maxillary sinus floor or, if the lesion encroached on the maxillary sinus, the centre of the lesion was below the level of the sinus floor. All other lesions were considered non-odontogenic.

**Table 1:** Clinical, radiological and histological parameters assessed, where possible, in ossifying fibroma cases

Clinical parameters	Radiological parameters	Histological parameters
Date of presentation Date of recurrence Gender Age at initial diagnosis Ethnic group Site Size Chief complaint Intra-oral examination	Anatomical site Maxilla Mandible, Craniofacial Anatomical location Anterior Posterior Angle/ramus Maxillary sinus Size Relationship to the inferior alveolar canal (Mandibular lesions) Relationship to floor of maxillary sinus (Maxillary lesions) Periphery of lesion Corticated Well defined Poorly defined Radiodensity Radiolucent ;Radio-opaque Mixed Other findings Tooth displacement Root resorption Cortical expansion	Border of the lesion Encapsulated Well defined Merged Nature of stroma Vascularity Cellularity Collagen Pattern Secondary changes Aneurysmal bone cyst Multinucleated giant cells Pattern of calcification Trabecular Psammomatoid Lamellar bone Woven bone

### Histopathology Details

In all 74 cases a description of the histopathology of the lesions was available from the pathology reports. In addition, haematoxylin and eosin (H&E) stained sections were available for review in 69 cases (Fig. 1). The H&E stained slides were reviewed and assessed for the histological findings (Table 1). Stromal features were assessed subjectively and vascularity, cellularity and collagen content were scored as low, intermediate or high, as outlined in Table 2.

**Table 2:** Criteria used for assessment of stromal features in ossifying fibroma

	Vascularity	Cellularity	Collagen content
Low	Small discrete vascular spaces constituting less than 25% of the lesion	Loose myxoid connective tissue	Cellular with little collagen content
Intermediate	Small discrete vascular spaces constituting more than 25% and less than 50% of the stroma	Cellular stroma with discrete fibroblastic cells	A mixture of fibroblastic cells and collagen fibres
High	Small vascular spaces constituting more than 50% of the stroma or large cystic spaces filled with blood	A high number of tightly packed fibroblastic cells	Predominantly mature collagen fibres with low cellularity

In addition, a simple stereological point counting method was used to estimate the area of calcified material, as well as the proportion of calcifications that were trabecular or psammomatoid. Point counting was carried out on one representative section from each case, using an eyepiece graticule and at × 100 magnification. Three random fields were counted with a minimum of 126 points on each section (range: 126 to 11,466).

### Statistical Analysis

Statistical analysis was used to evaluate the significance of associations between age, site, odontogenic, non-odontogenic, psammomatoid and trabecular lesions.

*p *values were obtained using two approaches: Fisher’s exact test was used for associations between categorical variables. For associations involving a numerical and a categorical variable either Welch Two Sample t-test or Wilcoxon rank-sum test was used with groupings of two levels. For groupings larger than two, ANOVA was performed. The significance level was adjusted to mitigate for the large number of pairwise comparisons performed on small sets so that p-values greater than 0.003 were not deemed significant.

## Results

### Clinical Findings

A total of 74 patients were included in this study, of whom 66% (*n* = 49) were female and 34% (*n* = 25) were male (M: F 1:2).

#### Clinical Presentation

##### Symptoms

Information on clinical symptoms was only available for 34 patients. The main presenting complaint was an intra-oral swelling (79%, *n* = 27) which was usually painless (65%, *n* = 22). Only 5 patients reported the swelling to be painful (15%) and two patients reported the presence of pain alone (6%). Facial deformity associated with eye protrusion was reported as a symptom in one patient (3%). In four patients (12%) the lesion presented as an asymptomatic incidental finding.

##### Duration

Lesion duration was recorded in 15 cases and ranged from 6 months to 32 years. Eight cases (53.3%) were present for 6 months or less and only four recorded a duration of 5 years or longer.

##### Signs

Clinical examination findings were documented in 46 cases. Intra-oral examination found a hard bony expansile lesion in 83% of cases (*n* = 38). The extent of expansion was recorded in 19 cases. Buccal expansion alone (58%, *n* = 11) was more common than lingual/palatal expansion (10%, *n* = 2) or combined bucco-lingual/palatal expansion (32%, *n* = 6). Other clinical findings included gingival enlargement (4%, *n* = 2), proptosis of the eye and facial asymmetry (4%, *n* = 2). Tooth displacement was found in two cases and loosening of the related teeth in just one case. Three cases were incidentally found on imaging (7%, *n* = 3).

##### Anatomical Location

Anatomical location was available for all 74 cases. Fifty-four cases (73%) were located in the mandible and 18 (24%) in the maxilla. Only 2 cases (3%) were located in other craniofacial bones, in the mastoid process and zygomatic frontal suture regions.

##### Age at Presentations

**Fig. 2:**
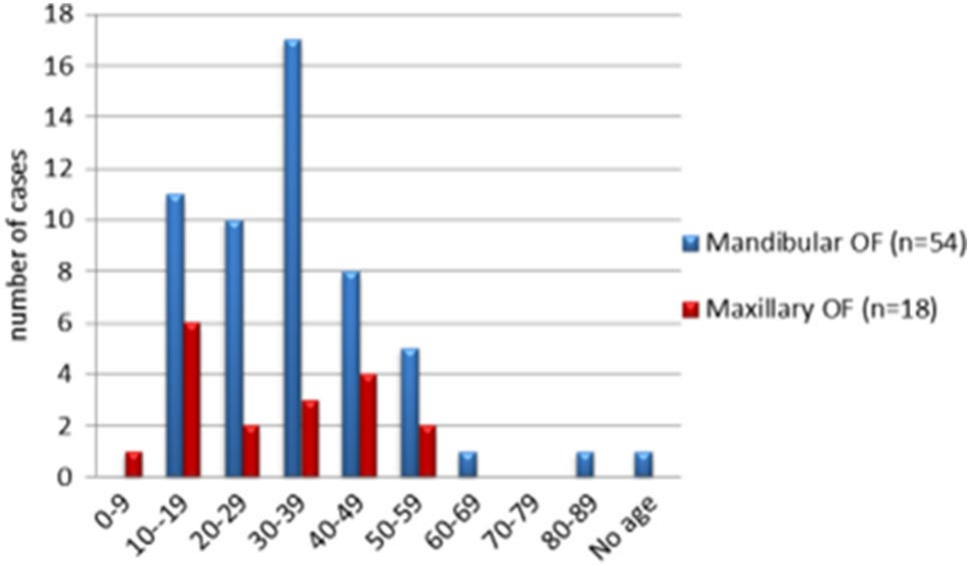
Comparison between the age distribution of mandibular and maxillary lesions. *P* value ˃ 0.03 (not significant)

Patient age was available for 73 cases. The average age of presentation at the time of diagnosis was 32.4 years, with a range of 6 years to 81 years. Overall, most cases arose in the second (23%, *n* = 17) and fourth (27%, *n* = 20) decades (Fig. 2). The peak of presentation for maxillary lesions was the second decade (33%, *n* = 6), whereas the peak age for mandibular lesions was in the fourth decade (31.5%, *n* = 17, Fig. 2).

**Fig. 3:**
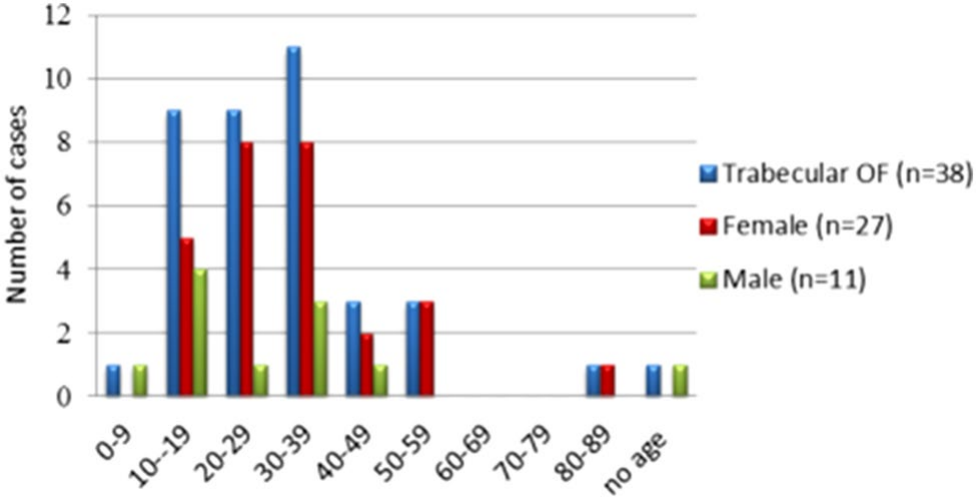
Distribution of trabecular OF by age and gender. Most of the cases were seen between second and fourth decade

**Fig. 4:**
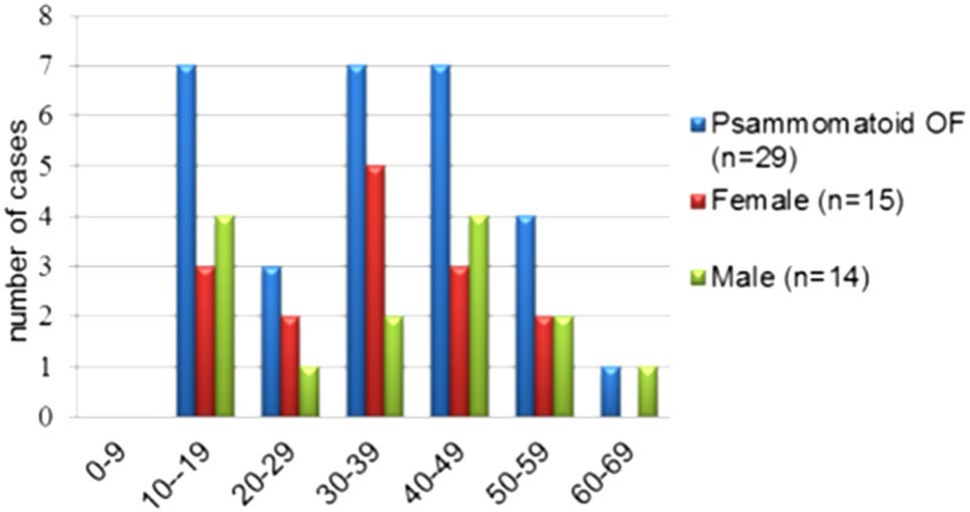
Distribution of psammomatoid OF by age and gender. The cases were equally distributed between second, fourth and fifth decade

Lesions classified as trabecular (*n* = 37) presented at an average age of 30 years (range 6–81 years) with the highest frequency across the second (24%, *n* = 9), third (24%, *n* = 9) and fourth decades (29%, *n* = 11) (Fig. 3). Lesions that were predominantly psammomatoid (*n* = 29) showed an average age of 35 years (range 10–68) with equal peaks of 7 (24%) cases in each of the second, fourth and fifth decades (Fig. 4).

#### Radiographic Findings

**Fig. 5:**
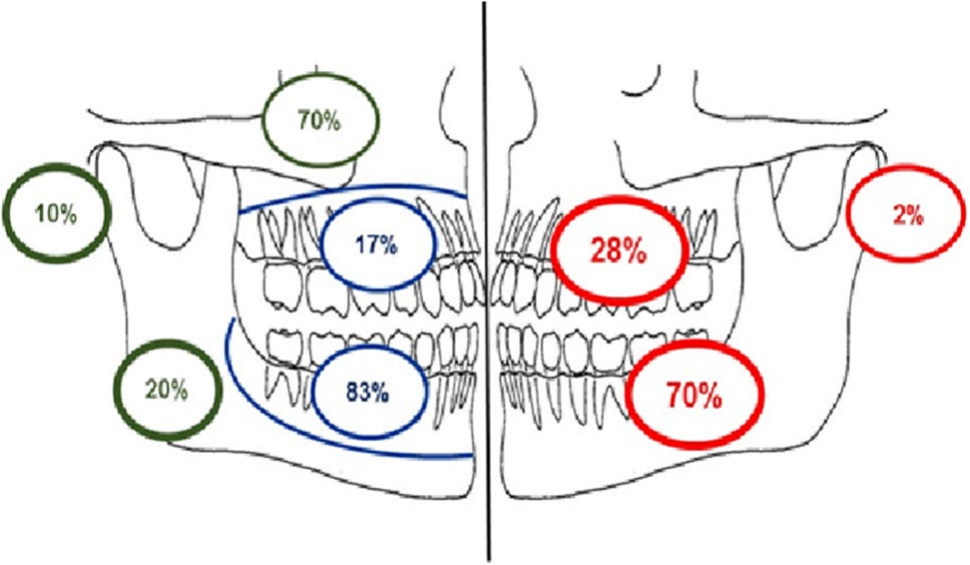
Site distribution of ossifying fibroma determined from available radiographs (red). Site distribution of odontogenic and non-odontogenic lesions within the maxilla, mandible and other craniofacial bones (Odontogenic–blue, non-odontogenic–green)

Radiographs were available for 46 cases. Figure 5 illustrates the distribution according to anatomical location and their designation as odontogenic or non-odontogenic. Thirty-two lesions (70%) were found in the mandible and 13 (28%) in the maxilla. One lesion was located in the mastoid process.

Table 3 illustrates the radiographic features of OF according to the extent of radiodensity, definition of border, degree of cortication and locularity. The majority of lesions (91%; *n* = 42) were unilocular and well defined, but only 30% had a corticated margin. Only 26 lesions (57%) showed a mixed pattern of radiodensity, while 17 (37%) were entirely radiolucent. Three cases (6%) were entirely radiopaque. In four cases of mixed radiolucency, the lesion was radio-opaque centrally and surrounded by a thin radiolucent margin. Cortical perforation was observed in one mandibular lesion and destruction of the lateral wall of the nose and floor of orbit was seen in one maxillary case. The average size of lesions was 29 mm (range: 7–75 mm). The mean size of mandibular lesions (29 mm) was larger than those presenting in the maxillary alveolus (14.2 mm), however, lesions in the maxillary sinus were, on average, the largest overall (40.8 mm).

**Table 3:** Summary of the radiographic features of ossifying fibroma

	Number (total = 46)	Percentage (%)
Radiodensity		
Mixed radiodensity	26	57
Radiolucent	17	37
Radiopaque	3	6
Border of lesion		
Well defined	42	91
Poorly defined	3	7
Variably defined	1	2
Cortication		
Not corticated	28	61
Corticated	14	30
Partly corticated	4	9
Locularity		
Unilocular	42	91
Multilocular	4	9

#### Histopathology

A description of the histological findings was available in the pathology reports for all 74 cases. Slides were available for review and stereological point counting in 69 cases, although the border of the lesion could be assessed in only 40 cases. Table 4 illustrates the histological features of OF according to definition of the border, stromal cellularity, vascularity and other features.

**Table 4:** Summary of the histological features of the ossifying fibroma

	Number (n)	Percentage (%)
Border of the lesion (total = 40)		
Circumscribed	24	60
Circumscribed with fibrous capsule	4	10
Completely merged	7	17.5
Locally merged	5	12.5
Cellularity of stroma (total = 74)		
Highly cellular	62	84
Moderately cellular	7	10
Low cellularity	1	1
Highly collagenous	3	4
Variable cellularity	1	1
Vascularity of stroma (total = 74)		
Highly vascular	6	8
Moderately vascular	8	11
Low vascularity	60	81
Other features		
MNGC	24	32
Chronic inflammatory cells	2	3
ABC-like features	3	4
Haemorrhage	3	4
Whorled pattern	14	19

*MNGC* Multinucleated giant cells, *ABC* Aneurysmal bone cyst

Based on the morphological descriptions in the pathology reports (*n* = 74) (Table 4), in the majority of cases the stroma comprised a hypercellular population of tightly packed spindled fibroblastic cells (84%, *n* = 62), with moderate (10%, *n* = 7) or low (*n* = 1) cellularity presenting much less commonly. Three cases (4%) exhibited a highly collagenous stroma and one case (1%) showed a variably cellular stroma with highly collagenous areas in a focal distribution (Table 4).

Vascularity was generally low (81%, *n* = 60), however, 8% (*n* = 6) of cases were highly vascular with 3 cases (4%) containing vascular pools surrounded by loose fibrous connective tissue resembling aneurysmal bone cyst-like features. Additional stromal findings included the presence of osteoclast-like multinucleated giant cells (MNGC) in 24 cases (35%) presenting as occasional scattered cells or as focal aggregates (Table 4).

**Fig. 6:**
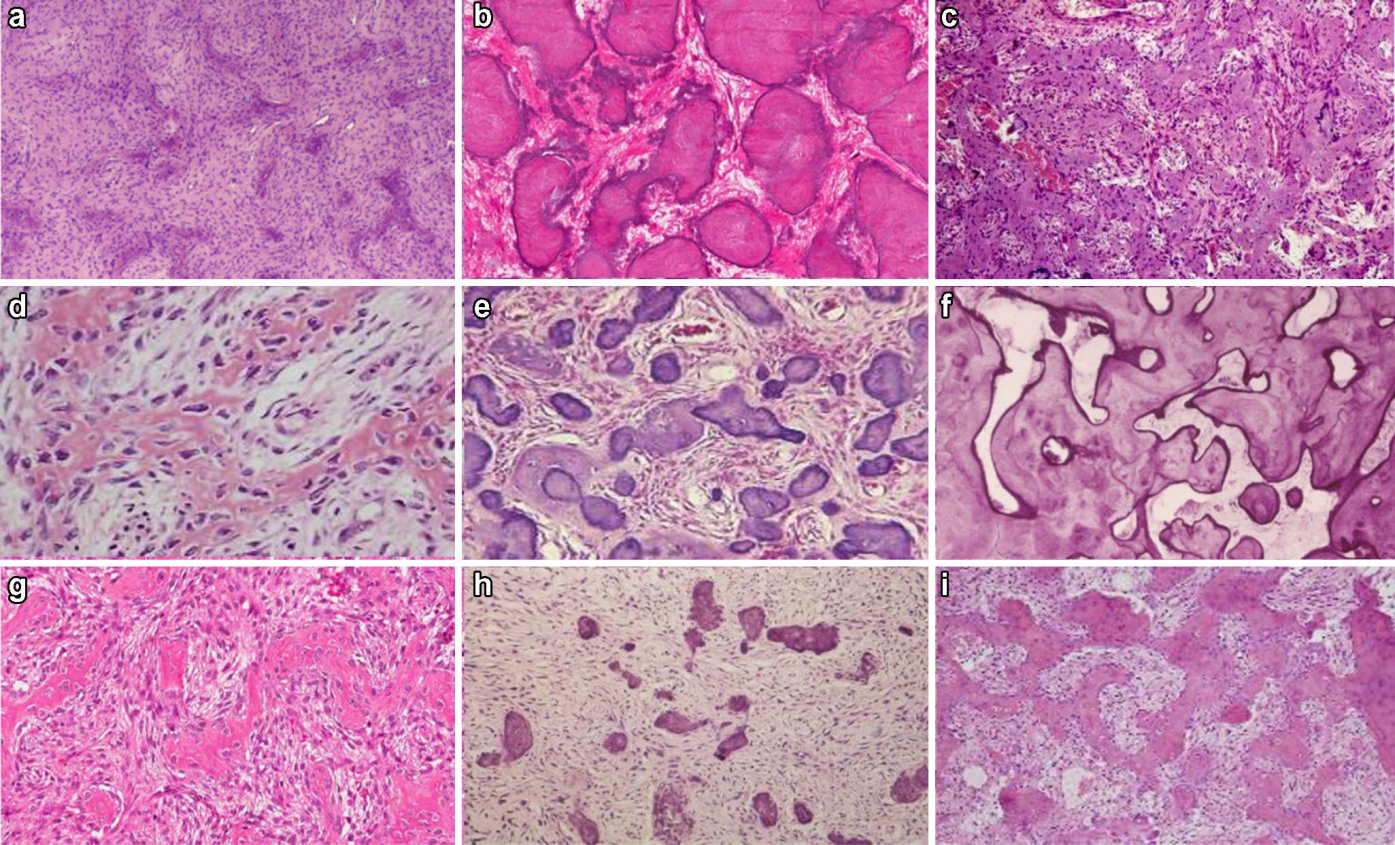
The varied morphological appearances of the mineralised component in OF including trabecular (**a**, **c**, **d**, **f**, **g**, **i**) and psammomatoid patterns (**b**, **e**, **h**)

The mineralised component showed a variable range of morphologic patterns including trabecular and psammomatoid patterns (Fig. 6). The bony trabeculae were of variable shape and size including thin or thick anastomosing strands, irregular bulbous trabeculae and trabeculae fused into large sheets. Occasionally, there was osteoblastic rimming surrounding the trabeculae***.*** Similarly, the psammoma-like bodies showed variable patterns ranging from well-formed acellular spherical masses to less well-formed irregular masses with basophilic centre and peripheral hyalinisation. Occasionally, the psammoma-like bodies showed feathery “wispy” outlines or fused to form irregular ‘ginger root’ patterns.

Sixty-nine cases with available histology slides were reviewed for the pattern and quantity of calcifications. Twenty cases (29%) exhibited an exclusively psammomatoid pattern of calcification, and 17 (25%) had a trabecular pattern only. The remainder (*n* = 32; 46%) showed a mixed pattern of trabecular and psammomatoid calcification, but in most cases one or other pattern predominated. In particular, 11 cases were predominantly trabecular but with up to 10% of calcifications showing a psammomatoid pattern. When classified by the predominant pattern (> 70% component), 37 cases (55%) were classified as trabecular and 29 (41%) were psammomatoid. Three cases were mixed, but in these, the psammomatoid element was slightly higher (55% to 69%).

Stereological point counting (*n* = 69) showed that the majority of lesions (*n* = 63) showed a high proportion (60% or greater) of stroma, with 35% (*n* = 24) comprising 90% or more stroma. In comparison to the stroma, the mineralised component accounted for less than 50% in most cases (96%; *n* = 66). In 6 cases, the calcified tissue comprised less than 1.0% of the lesion (range: 0.04% to 0.89%). In the lesions classified as psammomatoid, the mean proportion of stroma and calcification was 84% and 16%, respectively, compared to 78% and 22% in the trabecular lesions. However, this difference was not significant (*p* > 0.03).

#### Comparison of Juvenile and Adult Lesions

Cases were separated into either juvenile (< 18 years) and adult (≥ 18 years) based on age. The gender ratio was closer to equal in the paediatric population (M: F: 1:1.3) compared to the female predominance seen in the adult population (M: F 1:2.2). No statistical differences in age distribution were found for site, clinical or radiological presentation or histological pattern (Table 5). Lesions in adults were statistically more likely to be composed of woven bone only (*p* = 0.006), and proportionally more juvenile lesions displaying osteoid (Table 5).

**Table 5:** Comparison of features between paediatric and adult lesions

	No. (%)		
	Paediatric (< 18)	Adult (> 18)	*p* value
	14 (18.9%)	60 (81.1%)	
Mean age (*n* = 73)	12.7 years (6–16)	37.1 years (18–81)	
Gender (*n* = 74)			0.532
Male	6 (8.1)	19 (25.7)	
Female	8 (10.8)	41 (55.4)	
M:F	1:1.3	1:2.2	
Mean duration (*n* = 15)	6 months	57.4 months	0.324
Clinical presentation (*n* = 34)			*
Painless swelling	5 (14.7)	18 (52.9)	
Painful swelling	0 (0)	5 (14.7)	
Pain only	0 (0)	2 (5.9)	
No symptoms	1 (2.9)	3 (8.8)	
Site (*n* = 70)			0.336
Maxilla	6 (8.6)	12 (17.1)	
Mandible	8 (11.4)	43 (61.4)	
Other facial bones	0	1 (1.4)	
Mean size (*n* = 45)	30.6 mm	27.1 mm	0.571
Radiographic appearance (*n* = 46)			0.878
Radiopaque	0 (0)	3 (6.5)	
Radiolucent	5 (10.9)	13 (28.3)	
Mixed radiolucency	6 (13.0)	19 (41.3)	
Radiographic border (*n* = 46)			0.317
Well defined	11 (23.9)	30 (65.2)	
Poorly defined	0 (0)	5 (10.9)	
Cortication (*n* = 46)			0.306
Corticated	7 (15.2)	15 (32.6)	
Not corticated	4 (8.7)	20 (43.5)	
Odontogenic/Non-odontogenic (*n* = 46)			> 0.030
Odontogenic	8 (17.4)	28 (60.9)	
Non-odontogenic	3 (6.5)	7 (15.2)	
Histological pattern (*n* = 69)			0.880
Trabecular	9 (13.0)	29 (42.0)	
Psammomatoid	5 (7.2)	23 (33.3)	
Mixed	0 (0)	3 (4.3)	
Multinucleated giant cells (*n* = 69)	7 (10.1)	17 (24.6)	0.202
Presence of ABC-like features (*n* = 69)	1 (1.4)	2 (2.9)	0.472
Bone composition (*n* = 72)			**0.006**
Woven bone only	5 (6.9)	40 (55.6)	
Mixed woven and lamellar bone	3 (4.2)	13 (18.1)	
Mixed woven bone and osteoid	6 (8.3)	5 (6.9)	

^*^Insufficient data to compute *p* value. Significant values are indicated in bold

#### Comparison of Maxillary, Mandibular and Other Facial Bone Lesions

No statistically significant differences in clinical, radiological or histological features were found between maxillary, mandibular or other facial bone lesions (Table 6).

**Table 6:** Comparison of features between maxillary, mandibular and other facial bone lesions

	No (%)			
	Maxilla	Mandible	Other facial bones	*p* value
Mean age (*n* = 73)	30.3 years (6–54)	32.6 years (11–81)	27 years (27)	0.819
Gender (*n* = 74)				0.620
Male	8 (10.8)	17 (23.0)	0 (0)	
Female	10 (13.5)	34 (45.9)	1 (1.4)	
M:F	1:1.25	1:2	0:1	
Mean duration (*n* = 15)	6 months	27 months	-	0.746
Clinical presentation (*n* = 34)				*
Painless swelling	4 (11.8)	16 (47.0)	-	
Painful swelling	1 (2.9)	4 (11.8)	-	
Pain only	1 (2.9)	1 (2.9)	-	
No symptoms	1 (2.9)	3 (8.8)	-	
Mean size (*n* = 45)	18.7 mm	27.9 mm	18.0 mm	0.840
Radiographic appearance (*n* = 46)				0.306
Radiopaque	1 (2.2)	2 (4.3)	0 (0)	
Radiolucent	7 (15.2)	11 (23.9)	0 (0)	
Mixed radiolucency	4 (8.7)	20 (43.5)	1 (2.2)	
Radiographic border (*n* = 46)				1.000
Well defined	11 (23.9)	29 (63.0)	1 (2.2)	
Poorly defined	1 (2.2)	4 (8.7)	0 (0)	
Cortication (*n* = 46)				1.000
Corticated	6 (13.0)	16 (34.8)	0 (0)	
Not corticated	6 (13.0)	17 (37.0)	1 (2.2)	
Odontogenic/Non-odontogenic (*n* = 46)				> 0.030
Odontogenic	5 (10.8)	31 (67.4)	0 (0)	
Non-odontogenic	7 (15.2)	2 (4.3)	1 (2.2)	
Histological pattern (*n* = 69)				0.878
Trabecular	9 (13.4)	27 (40.3)	1 (1.5)	
Psammomatoid	9 (13.4)	19 (28.4)	0 (0)	
Mixed	0 (0)	2 (3.0)	0 (0)	
Multinucleated giant cells (*n* = 69)	4 (5.7)	17 (24.3)	1 (1.4)	0.273
Presence of ABC (*n* = 69)	0 (0)	2 (2.9)	0 (0)	1.000
Bone composition (*n* = 72)				0.250
Woven bone only	9 (13.0)	34 (49.3)	0 (0)	
Mixed woven and lamellar bone	5 (7.2)	9 (13.0)	1 (1.4)	
Mixed woven bone and osteoid	4 (5.8)	7 (10.1)	0 (0)	

^*^Insufficient data to compute *p* value

#### Comparison of Trabecular and Psammomatoid Lesions

**Fig. 7:**
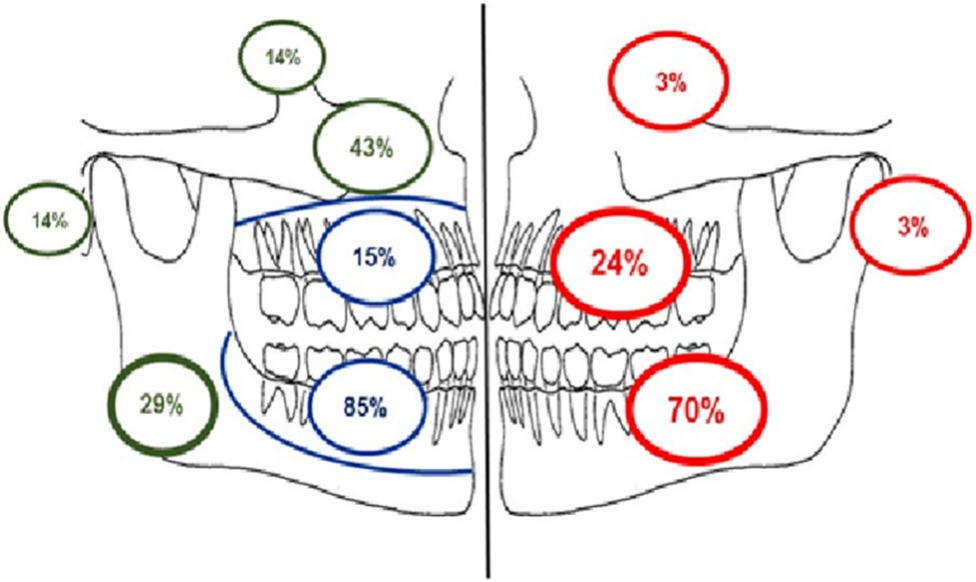
Distribution of all trabecular ossifying fibroma by location in percentage as determined by available radiographs (red). Distribution of odontogenic and non-odontogenic trabecular OF in mandible, maxilla and mastoid process (Odontogenic—blue, non-odontogenic—green)

Trabecular lesions (*n* = 38) mirrored that of the overall data, with a clear female predominance (M: F 1: 2.8) (Table 7). Figure 7 illustrates the distribution of trabecular lesions according to anatomical location showing that the majority (*n* = 27; 73%) were located in the mandible, 85% of which were classified as odontogenic. Indeed, 77% of all trabecular lesions were classified as odontogenic (*n* = 20) with only 6 (23%) judged to be non-odontogenic (Fig. 7).

**Table 7:** Comparison of features between trabecular and psammomatoid lesions

	No (%)			
	Psammomatoid	Trabecular	Mixed	*p* value
Mean age (*n* = 69)	35.1 (10–68)	30.3 (6–81)	26 (18–42)	0.361
Gender (*n* = 69)				0.136
Male	14 (20.3)	10 (14.5)	1 (1.4)	
Female	14 (20.3)	28 (40.6)	2 (2.9)	
M:F	1:1	1:2.8	1:2	
Mean duration (*n* = 15)	56 months	44 months	–	0.588
Clinical presentation (*n* = 34)				*
Painless swelling	3 (9.4)	16 (50.0)	2 (6.2)	
Painful swelling	4 (12.5)	1 (3.1)	0 (0)	
Pain only	2 (6.2)	0 (0)	0 (0)	
No symptoms	1 (3.1)	3 (9.4)	0 (0)	
Site (*n* = 67)				0.878
Maxilla	9 (13.4)	9 (13.4)	0 (0)	
Mandible	19 (28.4)	27 (40.3)	2 (3.0)	
Other facial bones	0 (0)	1 (1.5)	0 (0)	
Mean size (*n* = 45)	28.3 mm	26.4 mm	75.0 mm	**0.0221**
Radiographic appearance (*n* = 43)				0.341
Radiopaque	1 (2.3)	2 (4.7)	0 (0)	
Radiolucent	9 (20.9)	8 (18.6)	0 (0)	
Mixed radiolucency	6 (14.0)	16 (37.2)	1 (2.3)	
Radiographic border (*n* = 46)				0.678
Well defined	15 (34.9)	22 (51.2)	1 (2.3)	
Poorly defined	1 (2.3)	4 (9.3)	0 (0)	
Cortication (*n* = 46)				1.000
Corticated	8 (18.6)	13 (30.2)	1 (2.3)	
Not corticated	8 (18.6)	13 (30.2)	0 (0)	
Odontogenic/Non-odontogenic (n = 44)				1.000
Odontogenic	13 (29.5)	20 (45.4)	1 (2.1)	
Non-odontogenic	4 (9.0)	6 (13.6)	0 (0)	
Multinucleated giant cells (*n* = 69)	3 (4.3)	18 (26.1)	2 (2.9)	0.001
Presence of ABC (*n* = 69)	1 (1.4)	0 (0)	1 (1.4)	0.037
Bone composition (*n* = 69)				**0.000001**
Woven bone only	27 (39.1)	14 (20.3)	2 (2.9)	
Mixed woven and lamellar bone	0 (0)	15 (21.7)	0 (0)	
Mixed woven bone and osteoid	1 (1.4)	9 (13.0)	1 (1.4)	

^*^Insufficient data to compute *p* value. Significant values indicated in bold

In contrast to both the overall female predominance and to that seen in trabeculae lesions, psammomatoid lesions (*n* = 28) showed an equal female (50%, *n* = 14)-to-male (50%, *n* = 14) ratio (M:F 1:1). In fact, 58% of all male cases showed a psammomatoid pattern, compared to only 33% of female cases.

**Fig. 8:**
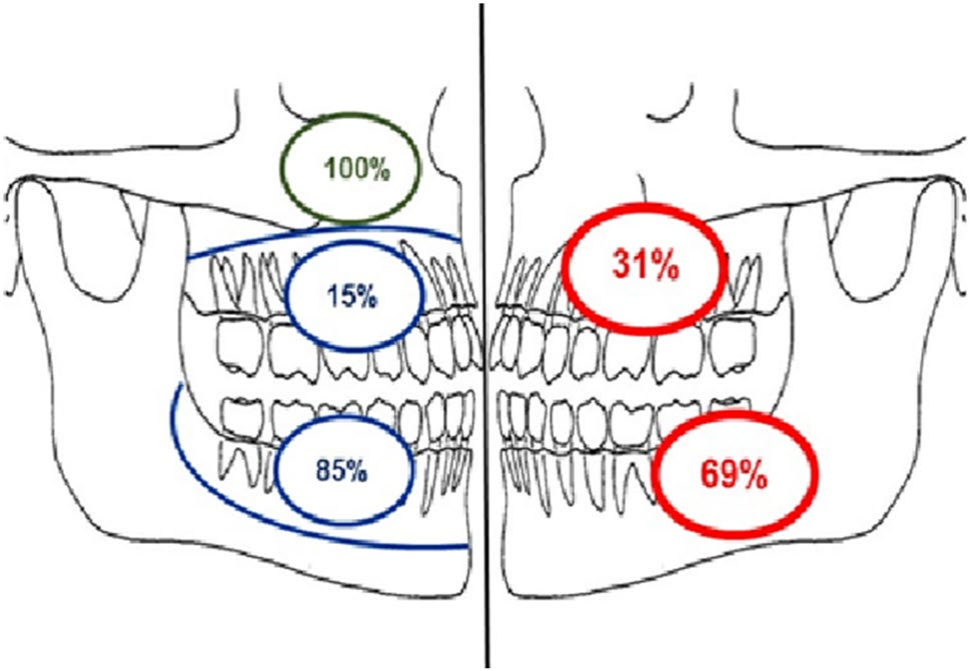
Distribution of all psammomatoid OF by location in percentage as determined by available radiographs (red). Distribution of odontogenic and non-odontogenic psammomatoid OF in mandible, maxilla (Odontogenic—blue, non-odontogenic—green)

Figure 8 illustrates the distribution of psammomatoid lesions (*n* = 28) according to anatomical location within the mandible and maxilla and their classification as odontogenic or non-odontogenic. 75% were odontogenic, of which 11 (85%) were located in the mandible. All non-odontogenic psammomatoid lesions were located in the maxillary sinus.

Lesions with a mixed trabecular and psammomatoid pattern are statistically larger in size (*p* = 0.02). Trabecular lesions showed mixed bone compositions from osteoid, to woven and lamellar bone. Psammomatoid lesions were almost exclusively comprised of woven bone only (96%, *n* = 26, *p* = 0.000001).

#### Comparison of Odontogenic and Non-Odontogenic Lesions

Overall, 36 (78%) lesions were classified as odontogenic, of which 83% (*n* = 30) were located in the mandible and 17% (*n* = 6) in the maxilla. Ten lesions (22%) were non-odontogenic, seven of which were found associated with the maxillary sinus (70%). Two were found in the posterior mandible (20%) and one in the mastoid process (10%).

Odontogenic lesions (*n* = 36) were seen at an average age of 30.2 years (range: 10–81 years) and showed peaks in the second (28%, *n* = 10) and fourth (33%, *n* = 12) decades. In comparison, non-odontogenic OF (*n* = 10) were seen at a slightly younger average age of 27.5 years (range 6–54 years) with a peak in the second decade (30%, *n* = 3).

Odontogenic lesions were statistically more common in the mandible (*n* = 30: 65%. *p* = 0.0001) (Table 8). No statistical differences were found between odontogenic and non-odontogenic lesions for other clinical or radiological presentations or histological pattern (Table 8).

**Table 8:** Comparison of features between odontogenic and non-odontogenic lesions

	No (%)		
	Odontogenic	Non-odontogenic	*P *value
Mean age (*n* = 46)	30.2 years (10–81)	27.5 years (6–54)	0.636
Gender (*n* = 46)			1.000
Male	13 (28.3)	4 (8.7)	
Female	23 (50.0)	6 (13.0)	
M:F	1: 3.8	1: 3.3	
Mean duration (*n* = 15)	210 months	30 months	*
Clinical presentation (*n* = 18)			*
Painless swelling	12 (66.7)	1 (5.6)	
Painful swelling	3 (16.7)	0 (0)	
Pain only	1 (5.6)	0 (0)	
No symptoms	1 (5.6)	0 (0)	
Site (*n* = 46)			**0.0001**
Maxilla	6 (13.0)	7 (15.2)	
Mandible	30 (65.2)	2 (4.3)	
Other facial bones	0 (0)	1 (2.2)	
Mean size (*n* = 45)	26.0 mm	34.9 mm	0.123
Radiographic appearance (*n* = 46)			0.201
Radiopaque	1 (2.2)	2 (4.3)	
Radiolucent	15 (32.6)	3 (6.5)	
Mixed Radiolucency	20 (43.5)	5 (10.9)	
Radiographic border (*n* = 46)			1.000
Well defined	32 (69.6)	9 (19.6)	
Poorly defined	4 (8.7)	1 (2.2)	
Cortication (*n* = 46)			0.725
Corticated	18 (39.1)	4 (8.7)	
Not corticated	18 (39.1)	6 (13.0)	
Histological pattern (*n* = 43)			1.000
Trabecular	20 (46.5)	6 (14.0)	
Psammomatoid	12 (27.9)	4 (9.3)	
Mixed	1 (2.3)	0 (0)	
Multinucleated giant cells (*n* = 46)	10 (21.7)	4 (8.7)	0.464
Presence of ABC (*n* = 46)	1 (2.2)	0 (0)	1.000
Bone composition (*n* = 45)			0.883
Woven bone only	21 (46.7)	5 (11.1)	
Mixed woven and lamellar bone	7 (15.6)	3 (6.7)	
Mixed woven bone and osteoid	7 (15.6)	2 (4.4)	

^*^Insufficient data to compute *p* value. Significant values indicated in bold

## Discussion

This review highlights the wide age distribution of OF, with cases presenting between 6 and 81 years, and peaks of presentation between the second and fourth decades. This wide age range is consistent with the previous studies [10, 20–22]. A younger age distribution has been reported by Johnson et al. [23] who reported an OF lesion in a 3-month-old child.

OF showed a female predilection with ratio of 2:1. Although, this female preference was much higher (5:1) in a case series presented by Eversole et al. [20], other authors state that OF showed only a slight female predilection [22, 24, 25]. In our study, the female predilection was present in all age groups except in young patients under 10 years of age. Of particular interest is that odontogenic lesions in patients under 30 years old were almost exclusively in females. In their retrospective case series, Liu et al*.* found a male predominance in those under the age of 18 [26].

Clinically, the lesions usually presented as painless swellings with no other accompanied symptoms, but rarely pain, soreness, sinus discharge, facial deformity and eye protrusion have been reported. Waldron, [27] stated that small OFs rarely presented clinically and were usually detected during routine radiographic examination, while larger lesions presented with swelling but rarely with pain. In our study, pain was the second most common presenting complaint, but this was still observed in only 18%. Similar results were observed by Sopta et al. [28]. MacDonald-Jakowski and Li, in their Hong Kong series, found that lesions associated with pain were seen in patients who were significantly older [10].

The duration of lesions varied from six months to 32 years. Rapid growth or bone destruction was not reported. In our study, the lesions with the longest reported duration were seen in the mandible and the zygomatic lesion. This longer evolution may be related to the nature of the bone at these sites compared to the cancellous bone of the maxilla. In a review conducted by El-Mofty et al. [29], large mandibular OF tended to expand inferiorly, and perforation of the cortical plates was rare and reported in only one case. The most likely explanation of these findings was that the slow growing rate of the lesions allowed reactive new bone formation at the periphery, rather than resulting in osteolysis.

Radiologically, OF commonly present as a radiolucent lesion containing focal or scattered radio-opacities, further supported by the findings of this study. Nearly all lesions were well defined on radiographs, and poorly defined borders were only seen in 4 cases, all of which were in the mandible, and were less than 20 mm in diameter.

Many previous studies reported that tooth displacement was often associated with jaw lesions [5, 30] with no root resorption [31]. Based on our observations, we found that OF can occasionally cause root resorption of the associated teeth, but this was seen in only four cases, mostly affecting the first molar tooth of the mandible. Although rare, MacDonald-Jankowski states that root resorption is still more frequent in cemento-ossifying fibroma than in focal cemento-osseous dysplasia and is one of the few helpful radiographic features in diagnosis [22].

Our findings agree with McCarthy [32] and suggest that the radiological features cannot be used to differentiate OF from other fibro-osseous lesions, as OF can grow to a large size and poorly or variably defined borders are seen in up to 9% of cases (*n* = 4, Table 3), similar to the features seen with fibrous dysplasia, which may also show more diffuse enlargement of the bone.

As far as we are aware, this study is the first to use a point counting (SPC) method, to estimate the proportion of stroma and calcifications within lesions. Consistent with the previous studies [11, 20], we found that the ratio of stroma to calcification to be markedly varied. SPC was used to classify OF into trabecular and psammomatoid lesions and to compare these histological variants. The comparison of both lesions revealed no significant differences between the variants with regard to demographic or site distribution. An interesting finding was the presence of a slight male predilection (M: F = 4:3) of psammomatoid lesions in the patients in the second decade. A similar slight male predilection was also observed by Makek [17] and Wenig et al. [33].

Our analysis demonstrated no statistical differences between the odontogenic and non-odontogenic lesions in terms of age, gender, size or histological features, however, we acknowledge that given the low number of craniofacial cases these data are likely to be skewed. Nevertheless, lesions in both the tooth-bearing and non-tooth-bearing areas can exhibit a trabecular or psammomatoid pattern of calcification.

In general terms, we can consider the ‘classical’ presentation of ossifying fibroma to be a slow growing, well demarcated, painless swelling affecting patients over a large age range with a predilection towards females. These findings, in particular the radiology, are often the most helpful when differentiating ossifying fibroma from other fibro-osseous lesions. However, this study shows that we also see great variation in all clinical, radiological and histological aspects of OF and that no one feature can be used as pathognomonic.

Additionally, based on these data, sub-classification of ossifying fibroma is challenging due to extensive overlap in both clinical and histological features; raising the question of whether distinct variants should exist at all. Arguably, this study shows few statistically significant findings in relation to age, site, histological pattern or odontogenic versus non-odontogenic origin to support the current classification. Despite this, there remains a shift of the data with regard to paediatric, male, maxillary, psammomatoid cases.

From this study, alterations to the classification of JTOF and POF variants cannot be made, given the limited number of paediatric and craniofacial cases, together with lack of recurrence data. However, unless further clinical or prognostically significant findings can be shown, the authors suggest that the classification of these lesions be changed to more accurately reflect the diversity of these lesions. The use of the term ‘cemento-’ has little diagnostic meaning and could be made redundant, as similar morphological features are seen both within the tooth-bearing areas of the jaws and elsewhere in the craniofacial skeleton, and that ‘ossifying fibroma’ is a better encompassing term to reflect all OF’s with conventional histopathological features.

## Conclusion

OF is a benign fibro-osseous tumour that encompasses a heterogeneous group of lesions of the craniofacial skeleton that show variable clinical and microscopic features. Apart from site of occurrence, odontogenic and non-odontogenic variants of OF demonstrate no specific clinical features and both may show trabecular and/or psammomatoid patterns. Therefore, the classification of these lesions should be simplified to be more encompassing of the clinical variations seen in this broad group of lesions.

## Data Availability

Raw data available in Microsoft Excel upon request.

## Code Availability

R Core Team (2022). R: A language and environment for statistical computing. R Foundation for Statistical Computing, Vienna, Austria. URL: https://www.R-project.org/. Code available upon request.

## References

[1] Chi AC, Collins LHC (2022) Cemento-ossifying Fibroma. In: WHO Classification of Tumours Editorial Board. Head and Neck Tumours. International Agency for Research on Cancer; 2022. Section 7.4.2.4 (WHO classification of tumours series (5^th^ Edition) Vol. 9). Lyon (France). https://publications.iarc.fr/Book-And-Report-Series/Who-Classification-Of-Tumours

[2] Chi AC, Collins LHC (2022) Juvenile trabecular ossifying fibroma. In: WHO Classification of Tumours Editorial Board. Head and Neck Tumours. International Agency for Research on Cancer; 2022. Section 7.4.2.6 (WHO classification of tumours series (5^th^ Edition) Vol. 9). Lyon (France). https://publications.iarc.fr/Book-And-Report-Series/Who-Classification-Of-Tumours.

[3] Chi AC, Collins LHC (2022) Psammomatoid ossifying fibroma. In: WHO Classification of Tumours Editorial Board. Head and Neck Tumours. International Agency for Research on Cancer; 2022. Section 7.4.2.5 (WHO classification of tumours series (5^th^ Edition) Vol. 9). Lyon (France). https://publications.iarc.fr/Book-And-Report-Series/Who-Classification-Of-Tumours.

[4] Waldron CA, Giansanti JS (1973). Benign fibro-osseous lesions of the jaws: A clinical-radiologic-histologic review of sixty-five cases: Part II. Benign fibro-osseous lesions of periodontal ligament origin. Oral Surg, Oral Med, Oral Pathol.

[5] Su L, Weathers DR, Waldron CA (1997). Distinguishing features of focal cemento-osseous dysplasias and cemento-ossifying fibromas: I. A pathologic spectrum of 316 cases. Oral Surg, Oral Med, Oral Pathol, Oral Radiol, Endodontol.

[6] Su L, Weathers DR, Waldron CA (1997). Distinguishing features of focal cemento-osseous dysplasia and cemento-ossifying fibromas: II. A clinical and radiologic spectrum of 316 cases. Oral Surg Oral Med Oral Pathol Oral Radiol and Endodontol.

[7] Kramer I, Pindborg J, Shear M (1992). WHO International Histological classification of tumours. Histological typing of odontogenic tumours.

[8] Barnes L, Eveson JW, Reichart P, Sidransky D (2005). Odontogenic tumours.Ch 6, WHO classification of tumors pathology and genetics of head and neck tumours.

[9] El-Naggar AK, Chan JKC, Grandis JR, Takata T, Slootweg PJ (2017). WHO classification of head and neck tumours chapter 8.

[10] MacDonald-Jankowski DS, Li TK (2009). Ossifying fibroma in a Hong Kong community: the clinical and radiological features and outcomes of treatment. Dentomaxillofacial radiol.

[11] Desai RS, Bansal S, Shirsat PM, Prasad P, Sattar S (2020). Cemento-ossifying fibroma and juvenile ossifying fibroma Clarity in terminology. Oral Oncol.

[12] Chang CC, Hung HY, Chang JYF, Yu CH, Wang YP, Liu BY, Chiang CP (2008). Central ossifying fibroma: a clinicopathologic study of 28 cases. J Formos Med Assoc.

[13] Speight PM, Carlos R (2006). Maxillofacial fibro-osseous lesions. Curr Diagn Pathol.

[14] Halkias LE, Larsen PE, Allen CM, Steinberg MJ (1998). Rapidly growing, expansile mass of the mandible in a 6-year-old boy. J Oral Maxillofac Surg.

[15] Nelson BL, Philips BJ (2019). Benign fibro-osseous lesions of the head and neck. Head Neck Pathol.

[16] El-Mofty SK (2002). Psammomatoid and trabecular juvenile ossifying fibroma of the craniofacial skeleton: two distinct clinicopathologic entities. Oral Surg, Oral Med, Oral Pathol, Oral Radiol, and Endodontol.

[17] Makek M (1983). Clinical pathology of fibro-osteo-cemental lesions of the cranio-facial skeleton and jaw bones.

[18] Slootweg PJ, Panders AK, Koopmans R, Nikkels PGJ (1994). Juvenile ossifying fibroma. An analysis of 33 cases with emphasis on histopathological aspects. J Oral Pathol Med.

[19] Zegalie N, Speight PM, Martin L (2015). Ossifying fibromas of the jaws and craniofacial bones. Diagn Histopathol.

[20] Eversole LR, Leider AS, Nelson K (1985). Ossifying fibroma: a clinicopathologic study of sixty-four cases. Oral surg, Oral Med, Oral Pathol.

[21] Urs AB, Kumar P, Arora S, Augustine J (2013). Clinicopathologic and radiologic correlation of ossifying fibroma and juvenile ossifying fibroma—an institutional study of 22 cases. Ann Diagn Pathol.

[22] MacDonald-Jankowski DS (2009). Ossifying fibroma: a systematic review. Dentomaxillofacial radiol.

[23] Johnson LC, Yousefi MEHDI, Vinh TN, Heffner DK, Hyams VJ, Hartman KS (1990). Juvenile active ossifying fibroma. Its nature, dynamics and origin. Acta Otolaryngol Suppl.

[24] Sciubba JJ, Younai F (1989). Ossifying fibroma of the mandible and maxilla: review of 18 cases. J Oral Pathol Med.

[25] Chang CC, Hung HY, Chang JYF, Yu CH, Wang YP, Liu BY, Chiang CP (2008). Central ossifying fibroma: a clinicopathologic study of 28 cases. J Formos Med Assoc.

[26] Liu YS, X-F., Guo, X-S., Xie, S., Cai, Z-G. (2017). Clinicopathological characteristics and prognosis of ossifying fibroma in the jaws of children: a retrospective study. J Cancer.

[27] Waldron CA, Neville BW, Damm DD, Allen CM, Bouquot JE (2002). Bone pathology. Oral and maxillofacial pathology.

[28] Sopta J, Drazic R, Tulic G, Mijucic V, Tepavcevic Z (2011). Cemento-ossifying fibroma of jaws-correlation of clinical and pathological findings. Clin Oral Invest.

[29] El-Mofty SK (2014). Fibro-Osseous Lesions of the Craniofacial Skeleton: An Update. Head Neck Pathol.

[30] Liu Y, Wang H, You M, Yang Z, Miao J, Shimizutani K, Koseki T (2014). Ossifying fibromas of the jaw bone: 20 cases. Dentomaxillofacial Radiol.

[31] Waldron CA (1985). Fibro-osseous lesions of the jaws. J Oral Maxillofac Surg.

[32] McCarthy EF (2013). Fibro-osseous lesions of the maxillofacial bones. Head Neck Pathol.

[33] Wenig BM, Vinh TN, Smirniotopoulos JG, Fowler CB, Houston GD, Heffner DK (1995). Aggressive psammomatoid ossifying fibromas of the sinonasal region. A clinicopathologic study of a distinct group of fibro-osseous lesions. Cancer.

